# Indentation fracture resistance of brittle materials using irregular cracks: A review

**DOI:** 10.1016/j.heliyon.2023.e19361

**Published:** 2023-08-22

**Authors:** Alireza Moradkhani, Valiollah Panahizadeh, Mohammad Hoseinpour

**Affiliations:** Faculty of Mechanical Engineering, Shahid Rajaee Teacher Training University, Tehran, Iran

**Keywords:** Fracture resistance, Irregular cracks, VIF method, Brittle materials, Biomaterials

## Abstract

The equation of the Vickers indentation fracture (VIF) proposed by Moradkhani *et al.* (J. Adv. Ceram. 2, 87–102 (2013)) has been used for the determination of indentation fracture resistance from indented area containing irregular and branch cracks formed around Vickers diamond indentation impressions. The equation is based on the concept that the surface of the micro-cracks is related to the depth of the micro-cracks. Results of the published experimental data have been compared and analyzed with similar and dissimilar methods in all different tested materials, and the accuracy, advantages and disadvantages have been presented and discussed. The results show that, in 90% of the performed tests, the accuracy of the equation is more than 90% compared to methods based on conventional fracture mechanics (CFM) and Vickers indentation fracture (VIF). The results show a high degree of accuracy to date, but the style and method of analysis need to be explained more clearly. Also, there is a requirement for a more comprehensive test range for other brittle materials.

## 1 Introduction

Resistance to fracture in brittle materials such as bioceramics and hard biological tissues is assessed by their *K*_IFR_ value [[1], [2], [3], [4]]. *K*_IFR_, defined as resistance to cracking, has correlations with wear resistance [[5], [6], [7], [8], [9], [10]].

Various techniques have been developed for measuring the fracture toughness in mode I/indentation fracture resistance (*K*_IC_/*K*_IFR_) of brittle materials. They are often classified into two groups: conventional fracture mechanics (CFM) and indentation fracture (IF) [3]. In CFM, the *K*_IC_/*K*_IFR_ value is determined firstly by creating and using notches, secondly by induced pre-cracks in the samples. Methods such as Chevron Notch Beam (CNB) [[11], [12], [13]], Single-Edge Pre-cracked Beam (SEPB) [13,14], Single Edge Notch Beam (SENB) [14,15], Single Edge V-notch Beam (SEVNB) [16,17], Single Edge Laser Notch Beam (SELNB) [18,19], Double Cantilever Beam (DCB) [[20], [21], [22], [23]], Wedge-insert Double Cantilever Beam (WDCB) [20,22,23], and End-Loaded Split (ELS) [24,25] are some of these techniques, and many of these methods have been used for many years to determine the *K*_IC_ or *K*_IFR_ of materials. In comparison, CFM is based on sharp indenters such as Vickers [[26], [27], [28], [29], [30]], Knoop [[31], [32], [33]], Berkovich [34,35], and Conical [36], which are applied to the sample in a certain period and amount of loading and are often used for brittle materials [34,36]. In general, the application of these techniques in the two groups varies according to the material, the manufacturing costs and testing of samples, and the required accuracy [3]. However, the use of the IF technique to determine the *K*_IFR_ of brittle materials, biomaterials, and hard biological tissues is more prevalent [26,29,30].

The IF group of techniques is classified such as Cube-Corner Indentation Fracture (CCIF) [26], Vickers crack opening displacement (VCOD) [31], interface indentation fracture (IIF) and Vickers indentation fracture (VIF) [37] tests. The need for less equipment and raw materials, lower cost, simplicity and high speed of sample preparation are some of the advantages of the IF method [28,37,38]. In the VIF technique, direct crack length measurements are often used around the indented areas in the sample, made by the Vickers diamond [26,[39], [40], [41]]. Usually, the *K*_IFR_ of the samples is determined by the crack length parameters, quality and hardness of materials, and the amount of applied load [26,31]. The relationships in this method are varied, and some studies have reported high accuracy [41,42] while others have detected many errors, even up to 48%, in them [[43], [44], [45], [46]].

In the present study, an effort has been made to provide an overview of fracture resistance determination techniques such as CFM and VIF to analyze the advantages and disadvantages of equation proposed by Moradkhani *et al*. [47], of the VIF technique. In this equation, the surface and thickness of micro-cracks created in a sample appear to improve the accuracy of the equation according to the cost parameter. Accuracy of the results obtained by the conventional fracture resistance and VIF techniques have been compared with the published literature for a range of composites. The results often show a high-reliability index of the equation and a low cost of sample testing. Therefore, by comparing the obtained results, ambiguities and strengths of the equation are mentioned and criticized to obtain a clear picture of fracture resistance.

## 2 Theoretical background

The emergence of the idea that it is possible to obtain *K*_IFR_ in brittle materials using IF was formed by Palmqvist [48] In the 1950s; however, it was ignored until the mid-1970s when Evans and Charles [49] applied the VIF technique for single crystal oxides to cemented carbides and presented functional relations and classified them into two models. Later, this technique quickly became popular due to the reduction of testing costs and was widely used for brittle materials [26,[29], [30], [31],^50^,^51^].

**Fig. 1:**
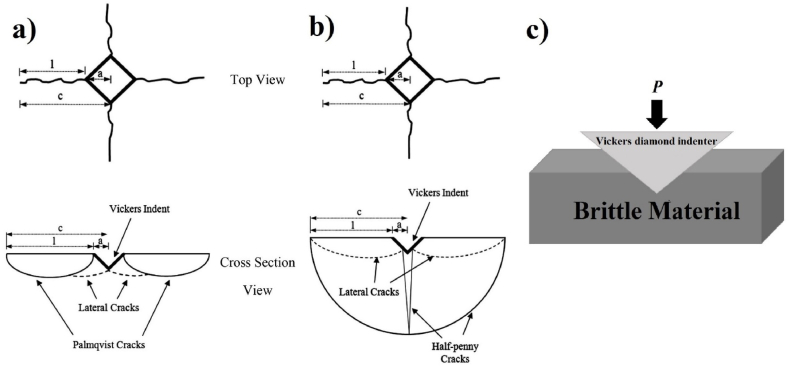
Schematic of a) Palmqvist crack model, b) Median/half penny crack model, and c) Vickers diamond indenter in brittle material.

In general, there are two models based on Palmqvist (*Pl*) and median/half-penny (*M*) cracks in the VIF technique [42,52]. Fig. 1 (a-b) shows differences between the two models, and Fig. 1c shows a schematic presentation of the Vickers diamond indenter in the sample. As can be seen, in the *Pl* model, the length of the formed cracks is considered only from the end of the indented area [53]. However, in the M-crack model, the length of the cracks is considered from the center of the indented section [42]. It is assumed [[54], [55], [56], [57]] that by increasing the Vickers diamond loading, the approach will change from the Palmqvist model to the median crack model. The results of some studies suggest that a simple way to separate these two models is sample surface polishing. After the polishing process, in the *Pl* model, the crack will be separated from the rhombic part left by the Vickers indenter; but in the median model, it will be connected to the remaining portion of the indenter [58,59]. Another way to distinguish between these two models is the ratio *c*/*a* of the width c of median crack to the half-width a of indenter diagonal length. If the ratio is *c*/*a* < 2.5, it is assumed as Palmqvist and if it is *c*/*a* ≥ 2.5, it will be considered an *M* crack. This ratio is often considered for brittle materials [60]. Some workers have considered 3 and 2 to separate the relationships in some materials and alloys [42,59].

After Evans and Charles [47,61], several relations have been proposed to calculate *K*_IFR_ [47,62,63], some of which are given in Table 1. As can be seen, Curve Fitting Technique (CFT) has been used in some relations. The basis of their formation is the use of trial and error and linking *K*_IFR_ to crack morphology [47]. Some of the relations in Table 1, relations 2, 3, 6, and 7, are simple to calculate *K*_IFR_ because they only depend on the parameters in Fig. 1. But some others, relations 9 and 10, depend on determining Vickers hardness in the sample. Finally, in other existing relations, including relations 1, 4, 5, 8, 11, 12, 13, and 14, in addition to the need to calculate the Vickers hardness, Young's modulus value of the samples must be calculated. Young's modulus of specimens is often measured based on the ASTM 769 [64] standard and the variation of sound velocity in the samples according to equation 1.

(1) $$E=\rho \;v^{2}$$

where *ρ* is sample density and *v* is the sample's sound velocity variation. This slows down the calculation process and increases the required equipment. But in these cases, *K*_IFR_ is more closely related to parameters dependent on the sample type and often increases accuracy [30,47,[65], [66], [67]].

**Table 1:** Some relations used in the calculation of fracture resistance (*K*_IFR_)/fracture toughness (*K*_IC_).

No.	Relation	Crack type	Author	Ref.
1	$K_{\text{IFR}}=0.018H_{V}\;a^{1/2}\;{\left(\frac{E}{H_{V}}\right)}^{2/5}\;{\left(\frac{c}{a-1}\right)}^{-1/2}$	*Pl*	Niihara	[68]
2	$K_{\text{IFR}}=0.0515\;\left(\frac{P}{c^{3/2}}\right)$	*Pl*	Lawn and Fuller	[69]
3	$K_{\text{IFR}}=0.079\;\left(\frac{P}{a^{3/2}}\right)\;\log\;\left(4.5\;\frac{a}{c}\right)$	*Pl*	Evans and Wilshaw	[70]
4	$K_{\text{IFR}}=0.015\;{\left(\frac{E}{H_{V}}\right)}^{2/3}\left(\frac{P}{c^{3/2}}\right)\;{\left(\frac{1}{a}\right)}^{-1/2}$	*Pl*	Lauger	[71]
5	$K_{\text{IFR}}=0.016\;{\left(\frac{E}{H_{V}}\right)}^{1/2}\frac{P}{C^{3/2}}$	*M*	Anstis *et al.*	[72]
6	$K_{\text{IFR}}=0.0752\frac{P}{C^{3/2}}$	*M*	Evans and Charles	[49]
7	$K_{\text{IFR}}=0.0726\;\frac{P}{C^{3/2}}$	*M*	Lawn and Fuller	[69]
8	$K_{\text{IFR}}=0.014\;{\left(\frac{E}{H_{V}}\right)}^{1/2}\frac{P}{C^{3/2}}$	*M*	Lawn *et al.*	[73]
9	$K_{\text{IFR}}=0\;.16\;H_{V}\;a^{1/2}\;{\left(\frac{c}{a}\right)}^{-3/2}$	*M*	Evans and Charles	[49]
10	$K_{\text{IFR}}=0.0889\;{\left(\frac{H_{V}.P}{\sum_{i=1}^{4}c_{i}}\right)}^{1/2}$	CFT	Shetty *et al.*	[54]
11	$K_{\text{IFR}}=0.4636{\left(\frac{E}{H_{V}}\right)}^{2/5}\frac{P}{a^{3/2}}10^{F}$	CFT	Evans	[55]
12	$K_{\text{IFR}}=0.018\;{\left(\frac{E}{H_{V}}\right)}^{1/2}\frac{P}{C^{3/2}}$	CFT	Japanese Standards Association	[56]
13	$K_{\text{IFR}}=\left(H_{V}a^{1/2}\right)\;{\left(\frac{E}{H_{V}}\right)}^{2/5}10^{y}$	CFT	Evans	[55]
14	$K_{\text{IFR}}=0.0782\;\left(H_{V}\;a^{1/2}\right)\;{\left(\frac{E}{H_{V}}\right)}^{2/5}\;{\left(\frac{c}{a}\right)}^{-1.56}$	CFT	Lankford	[57]

In Table 1, the dimensionless values of *F* and *y* in relations 11 and 13 in Table 1 are as follows [55]:

(2) $$F=-1.59-0.34x-2.02x^{2}+11.23x^{3}-24.97x^{4}+16.32x^{5}$$

(3) $$y=-1.59-0.34x-2.02x^{2}+11.23x^{3}-24.97x^{4}+15.32x^{5}$$

The value of *x* in equations (2), (3)) equals equation (4).

(4) $$x=\log\;\left(\frac{c}{a}\right)$$

## 3 Discussion

**Fig. 2:**
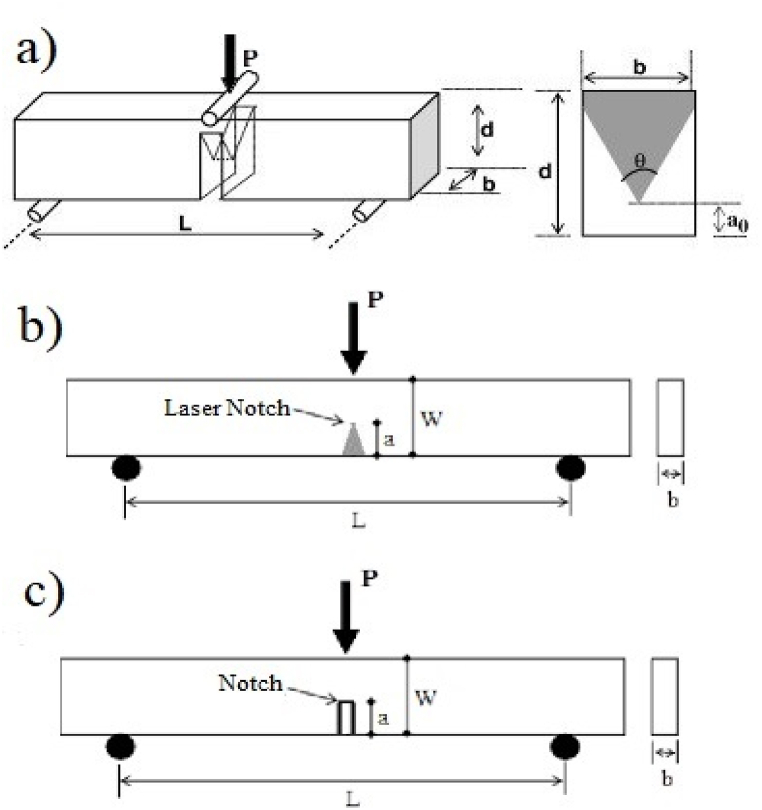
Schematic of a) Chevron Notch Beam (CNB), b) Single Edge Laser Notch Beam (SELNB), and c) Single Edge Notch Beam (SENB).

The presence of irregular and branched cracks in the samples causes errors in the *K*_IFR_ results obtained from the VIF relations and researchers have always tried to solve this issue [52,74]. The main reasons for these errors are high indentation loading and the presence of micro-cracks in the microstructure of the samples in brittle materials, which cause irregular cracks [72]. Even three-dimensional (3D) cracks and complexes for materials with ceramic coatings, formed in some composites, have been considered, and efforts have been made to resolve this issue [[75], [76], [77]]. One of these investigations was conducted by Moradkhani *et al*. [47] in 2013, in which they presented a semi-experimental equation (5) to minimize these errors. They believed that consideration of the mean penetration depth and effective area of formed micro-cracks and their involvement in the calculations would reduce the incidence of error. In addition, the depth and penetration of micro-cracks in the sample are directly related to the average surface and thickness of micro-cracks, and the involvement of these two parameters in the *K*_IFR_ calculation reduces the number of errors. On the other hand, this method significantly reduces testing costs because it prevents a large number of tests on the sample from achieving natural cracks [47,78]. Based on fitting the available data the following empirical equation was proposed [47]:

(5) $$K_{\text{IFR}}=0.00366\;{\left(\frac{E}{H_{V}}\right)}^{1/2}\;t_{\text{av}}^{3/2}\;\frac{P}{A^{3/2}}$$

where *t*_av_ is the average penetration depth of microcracks formed around the indented section (mm), and *A* is the surface area of the effect of the microcracks formed around the indented section (mm^2^). The coefficient in the equation is also obtained by performing mathematical and experimental calculations for Al_2_O_3_-nanoSiC nanocomposites with different volume percentages of nanoSiC. To determine this coefficient, the results of Single Edge Laser Notch Beam (SELNB) and Single Edge Notch Beam (SENB) methods have been used, and to ensure its value, the results of *K*_IFR_ have been compared with the Chevron Notch Beam (CNB) method. Fig. 2 (a-c) shows the schematics of three methods CNB, SELNB, and SENB to determine fracture toughness. Despite all three methods being highly accurate and having reliable results, the costs of making samples, especially for brittle materials and testing them, are always high [11,12,15,18]. As can be seen, the difficulty in making accurate samples leads workers to other methods such as Vickers indentation fracture [3].

Equation (5) is similar to relations 5, 8, and 12 of Table 1, which differ among themselves by the numerical factor equal to 0.016, 0.014, and 0.018, respectively. These relations follow from stress residual theory and the numerical factor in the case of half-penny cracks. According to this theory, the residual stress remaining around an indentation impression after removing the applied load on the indenter is the driving force for the creation of cracks [50,73]. Indentation pressure is produced by a disc-shaped indenter and can be calculated as *p* = *P*/A* in the surface area underneath the indenter, where *P* is the applied load and A* is the surface area underneath the indenter. An increase in indentation load is expected to result in an increase in the volume of the pressurized area, as calculated by *V* = A*h, where *h* is the penetration depth and A* is dependent on *A*, the area deformed around the indentation.

Assuming that the average depth *t* of cracks formed in the vicinity of indentation is equal to *h* one finds that both the depth *t* and the area *A* = *πC*^2^/4, where *C* is the diameter of the deformed area containing cracks [50]. This is the explanation of the inverse relation between *t* and *A*. For example, by comparing relation 8 of Table 1 with the constant 0.014 based on residual stress theory and equation (5), *t* and *C* can be expressed as *C* = 8.9*t*. However, it cannot be adopted for many emerging brittle materials, such as smart composites and biological hard tissues, which have complex microstructures.

**Fig. 3:**
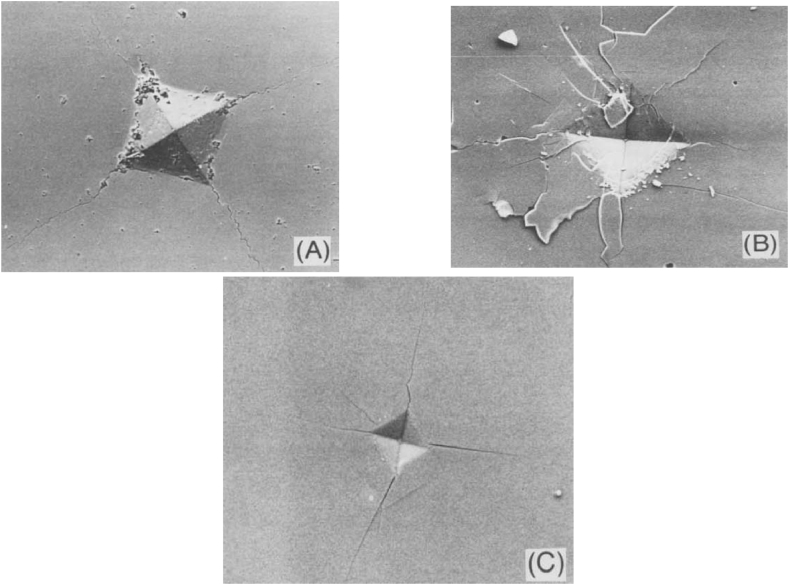
Scanning Electron Microscope of Al_2_O_3_ (A) indentation load 50 N, (B) 50 N; *Chipping* phenomenon, and (C) sapphire 10 N; branching cracks [72].

**Fig. 4:**
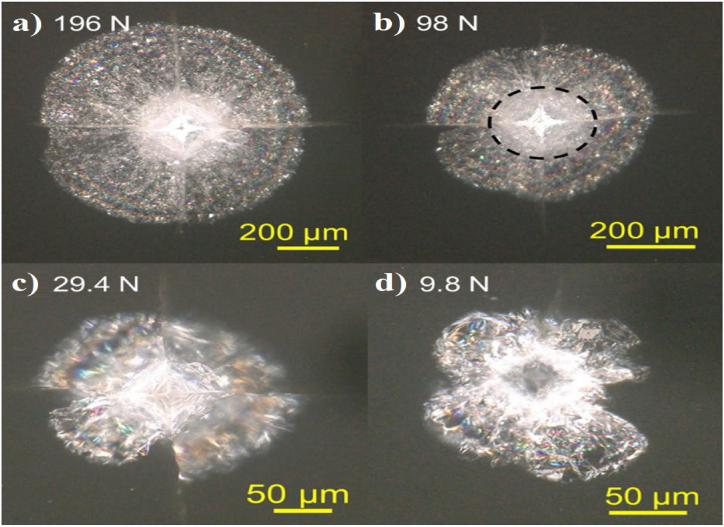
Optical micrograph of cracks induced by Vickers indentation fracture (VIF) in (CAS- GC) ceramics a) indentation load 196 N, b) 98 N, c) 29.4 N, and d) 9.8 N [77].

Moradkhani *et al.* [47] in their initial study, believed that the first condition for using equation (5) is the absence of *chipping* phenomenon in the samples. This phenomenon generally occurs at high loadings [72]. Also, the secondary condition is the minimum brittleness of the samples, which is achieved by establishing *nc*/*a* ≥ 20, *c*_*max*_ = 1.4*n*, if *n* ≤ 7 where *c* is the length of median crack (m), *a* is the diagonal half-length of Vickers impression (m), and *n* is the result of the number of cracks emitted from each side of the indented rhombus area. This claim indicates the non-necessity of having only four cracks without any branch, which distinguishes equation (5) from other relations of the same family and expands its efficiency in practice [47]. Fig. 3 shows the Scanning Electron Microscope (SEM) images of the residual effect of the Vickers indenter in Al_2_O_3_ samples. In Fig. 3a, the Vickers effect can be seen without branch cracks. In Fig. 3b, the loading value is so high that it causes *chipping* phenomenon in the sample, and Fig. 3c also contains branch cracks around the sections. Fig. 4 shows the Optical micrograph (OM) images of the residual effect of the Vickers indenter in CaO–Al_2_O_3_–SiO_2_ glass-ceramic (CAS-GC) samples at 196, 98, 29.4, and 9.8 N loads. Fig. 4a and b in return to smooth lateral cracks observed in the material. Four median cracks on the surface emanating from the corners of the indent. The micro-crack zone is defined as a circular opaque area at the center in the dashed line in Fig. 4b. In Fig. 4c and d the lateral crack system is less than or equal to 29.4 N [77].

**Fig. 5:**
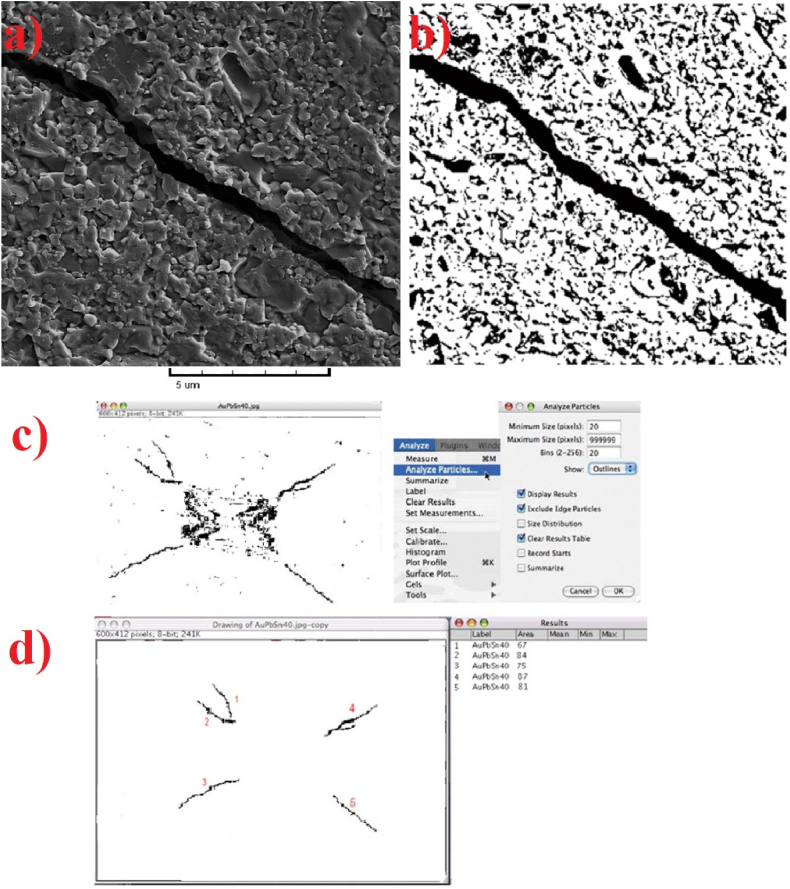
a) Formed crack in Al_2_O_3_–15%nanoSiC sample, b) preparation of the crack to be calculated by Image Analyzer Software, c) separation of cracks by software in order to measure the required values, and d) calculation the area values of cracks [47].

The Image Analyzer Software is commonly used to determine the parameters *t*_av_ and *A* based on the applied load to the diamond Vickers and the hardness values of the samples. For example, Fig. 5 (a-d) illustrates the process of using software to calculate these values for the formed cracks around the Vickers indenter in the Al_2_O_3_–15%nanoSiC sample [47]. In Fig. 5c and d, the software is being used to analyze and measure the images and area values of cracks, respectively.

**Fig. 6:**
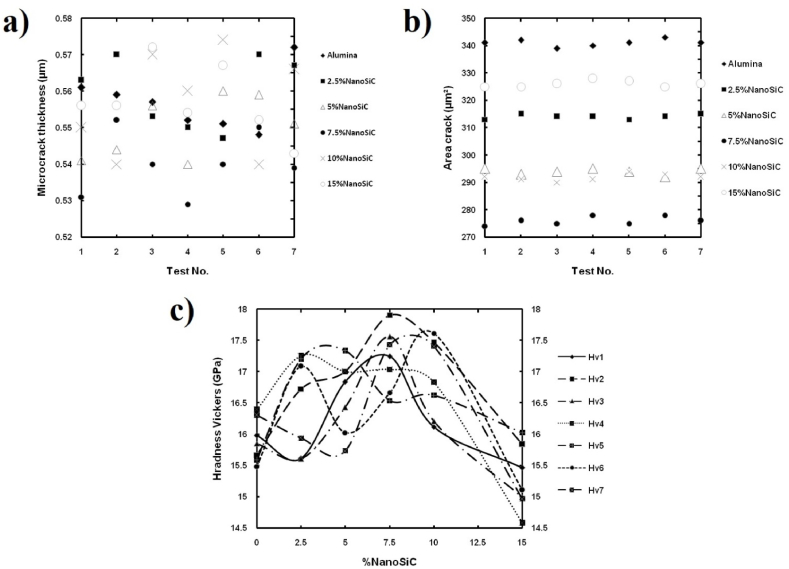
Measurement of values a) *t*_av_, b) *A*, and c) *H*_V_ in Al_2_O_3_ samples containing 0, 2.5, 5, 7.5, 10, and 15 vol% nanoSiC [47].

Fig. 6 (a-c) shows *t*_av_, *A*, and *H*_V_ values for calculating *K*_IFR_ in Al_2_O_3_ samples containing 0, 2.5, 5, 7.5, 10, and 15 vol% nanoSiC by measuring seven times. In this experiment, a 150 N force was applied to the samples for 15 s using an indentation test, and the thickness and area of microcrack tracks were measured [47]. However, the applied load values should be determined based on the hardness of the samples to create regular or irregular cracks without causing the *chipping* phenomenon, which allows for a wide range of applied loads to determine *t*_av_ and *A*.

In the following years, the accuracy and efficiency of equation (5) for determining fracture resistance in materials such as B_4_C-nanoTiB_2_ [79], B_4_C-nanoSiB_6_ [80], B_4_C-nanoTiB_2_-Fe/Ni [81], Al_2_O_3_–SiC [82], Al_2_O_3_–SiC–MgO [83], B_4_C-nano/microSiC [84], 3Y-TZP [85], Al_2_O_3_/3Y-TZP [86], W–ZrC [87], and Si particle [88] were tested. The mechanical and microstructural properties of these materials are diverse and have medical and industrial applications. Hence, the range of accurate measurement of the equation became relatively wide, but it seems that this equation is still not widely known and implemented.

**Fig. 7:**
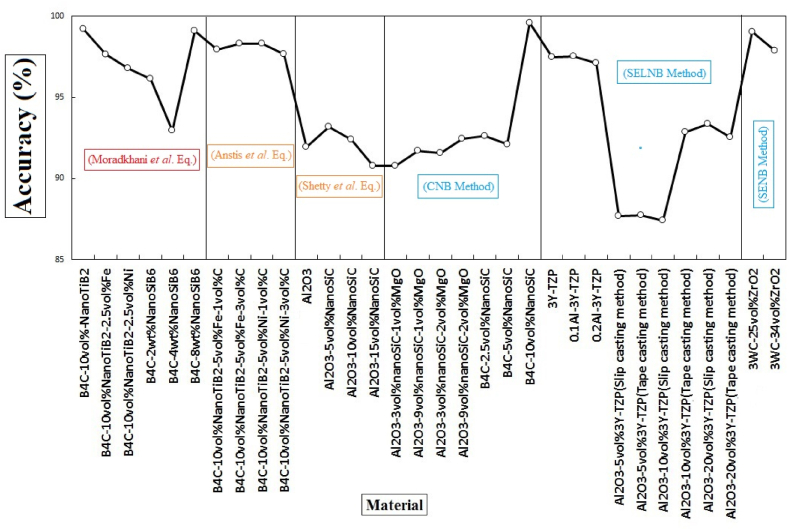
Comparison of the accuracy of the fracture resistance, Moradkhani *et al.* equation values with irregular cracks with other Vickers indentation fracture (VIF) relations and some conventional fracture mechanics (CFM) techniques in some brittle materials.

Fig. 7 compares the accuracy of *K*_IFR_ obtained from equation (5) for some of the materials made at their optimal sintering temperature. The comparison includes the most practical VIF relations mentioned in Table 1 (relations 5 and 10) and some conventional fracture mechanics techniques such as CNB, SELNB, and SENB methods [[79], [80], [81], [82], [83], [84], [85], [86], [87], [88]]. As can be seen, in 90% of the samples, more than 90% accuracy was reported. However, in some cases, such as Al_2_O_3_-3-YTZP composites made by tape/slip casting, the accuracy has been reduced to 87% compared to the SELNB method [86]. In some materials, the accuracy is close to 100% [80,84]. This accuracy has been observed in both CFM and VIF groups. From the results of articles published since the beginning of this equation, it seems that their accuracy ensures their reliability and no studies have been presented to refute them. In addition, equation (5) has been used by researchers in a wide range of other sintering temperatures as well as the various fabrication methods of the materials mentioned in Fig. 7, and similar accuracy has been reported for them [[79], [80], [81], [82], [83], [84], [85], [86], [87], [88]]. Considering these two parameters, a wide range of sintering temperatures and various fabrication methods, equation (5) has considered up to one hundred composites with other ranges of mechanical properties, especially *K*_IFR_. Despite the microstructural defects and other mechanical properties resulting from different fabrication methods and sintering temperatures, accuracy is still reported to be high.

It should be noted that the range of brittle materials with other properties and microstructures is much broader than the scope of research accuracy and can still challenge the accuracy of the equation. There seems to be a need for further exploration of the method, a more precise and more accurate presentation of how the test was performed, the tools and software used, and a more precise description of the mathematical calculations. Except for a limited number of studies, this equation has not been used. There is also an urgent need to test on standard materials with a wide range of *K*_IFR_. Some workers, such as Anstis *et al.* [72], did this process to establish the accuracy of their relation. Nevertheless, so far, despite testing and using the equation on a range of brittle materials, high accuracy in the output of the equation has been reported. This could lead to applying the equation in a broader range of brittle materials to reach a greater consensus among researchers.

Another critical point claimed in equation (5) is that there is no need to identify the type of Palmqvist/median crack model [80]. For example, in 3Y-TZP Dental Ceramics, the *c*/*a* ratio was calculated based on 29.42, 49.03, 147.09, 196.13, and 245.15 loading amounts, and this ratio ranged from 1.5 to nearly 4 for pure and silica-doped TZ, and 0.1/0.2A-TZ samples. Finally, the output of the equation for all ratios had an accuracy of over 90% compared to the relationships in Table 1 [85]. Also, in other studies, the amount of loading in B_4_C-nanoTiB_2_ samples was considered a variable parameter, and no effect on the accuracy of the equation was observed before *chipping* [89,90]. However, there seems to be a need for extensive research in this regard.

## 4 Summary and outlook

The present study reviews the published research results of the equation proposed by Moradkhani *et al.* [47] to estimate the *K*_IFR_ value using irregular and branch micro-cracks. It is based on measuring the *K*_IFR_/*K*_IC_ of brittle materials, at the lowest cost and, at the same time, the simplicity of the test steps [28]. In the last decade, ranges of brittle materials were tested with the aim of measuring the accuracy and benchmarking the accuracy of the equation outputs with various fabrication conditions, and satisfactory results were obtained [42]. However, so far, the scope of the practical application of this equation is limited to a few investigations [80]. There might be different reasons for its no widespread use; Failure to test it on known standard materials, duration and amount of loading [90], how exactly the crack effect level is determined [47], the probability of creating complex 3D cracks [77], lack of clear and accurate presentation of how the tests were performed and how the tools and software were used, and lack of accurate description of mathematical calculations and the existence of other different reliable relations [47,80]. However, so far, there have been no reports of inaccuracies in the equation outputs. In several studies, the accuracy for different materials has generally been more than 90% compared to CFM and VIF [[79], [80], [81], [82], [83], [84], [85], [86], [87], [88], [89], [90]]. However, there is a need for more theoretical and practical use which can prove its accuracy, this will reduce the cost of testing and could be a new approach to determining the *K*_IFR_/*K*_IC_. Moreover, sufficient attention should be paid to the range of test materials and other parameters that may be problematic during the tests.

In the last two decades, the theory of peridynamic has investigated the behavior and growth of cracks using mathematical models and simulations for brittle materials [[91], [92], [93], [94], [95]]. In this method, models and simulations explain the role Van der Waals forces play in the prediction of cracks behavior of brittle materials [[96], [97], [98], [99]]. The combination of the two methods, peridynamics theory and VIF [100,101], based on Griffith theory could be a new approach and a vast evolution in determining *K*_IFR_/*K*_IC_ for various materials, composites, and biological hard tissues.

## Author contribution statement

All authors listed have significantly contributed to the development and the writing of this article.

## Data availability statement

Data will be made available on request.

## Additional information

No additional information is available for this paper.

## Declaration of competing interest

The authors declare that they have no known competing financial interests or personal relationships that could have appeared to influence the work reported in this paper.

## Abbreviations used

CCIF: Cube-Corner Indentation Fracture
CFM: Conventional Fracture Mechanics
CFT: Curve Fitting Technique
CNB: Chevron Notch Beam
DCB: Double Cantilever Beam
ELS: End-Loaded Split
IF: Indentation Fracture
IIF: Interface indentation fracture
*K*_IC_: Fracture toughness in mode I
*K*_IFR_: Indentation fracture resistance
OM: Optical micrograph
SELNB: Single Edge Laser Notch Beam
SEM: Scanning Electron Microscope
SENB: Single Edge Notch Beam
SEPB: Single-Edge Pre-cracked Beam
SEVNB: Single Edge V-notch Beam
VCOD: Vickers crack opening displacement
VIF: Vickers Indentation fracture
WDCB: Wedge-insert Double Cantilever Beam

**Symbols**
*A*: Surface area of the effect of the micro-cracks formed around the indented section (mm^2^)
*A**: Surface area underneath the indenter (mm^2^)
*a*: Diagonal half-length of Vickers impression (m)
*C*: Diagonal length of a crack (m)
*c*: Length of median crack (m)
*E*: Young's modulus of the sample (GPa)
*H*_V_: Vickers hardness of the sample (GPa)
*h*: Penetration depth (m)
*l*: *c-a* (m)
*M*: Median/half penny
*P*: Vickers diamond loading (N)
*Pl*: Palmqvist
*p*: Indentation pressure (GPa)
*t*: Thickness of micro-cracks formed around the indented section (mm)
*t*_av_: Average penetration depth of microcracks formed around the indented section (mm)
*V*: Volume of area underneath indenter in disc-shaped (mm^3^)

## References

[1] Delage J., Saiz E., Al Nasiri N. (2022). Fracture behaviour of SiC/SiC ceramic matrix composite at room temperature. J. Eur. Ceram. Soc..

[2] Da D., Chen W. (2023). Simple strategy toward tailoring fracture properties of brittle architected materials. Int. J. Numer. Methods Eng..

[3] Bhattacharyya K. (2022). A comprehensive study of fracture toughness determination from conventional and unconventional methods. Defence Sci. J..

[4] Miqdad M., Syahrial A.Z. (2022). Effect of nano Al_2_O_3_ addition and T6 heat treatment on characteristics of AA 7075/Al_2_O_3_ composite fabricated by squeeze casting method for ballistic application. Evergreen.

[5] Gupta M.K., Singhal V., Rajput N.S. (2022). Applications and challenges of carbon-fibres reinforced composites: a Review. Evergreen.

[6] Jing Q., Bao J., Ruan F., Song X., An S., Zhang Y., Tian Z., Lv H., Gao J., Xie M. (2019). High-fracture toughness and aging-resistance of 3Y-TZP ceramics with a low Al_2_O_3_ content for dental applications. Ceram. Int..

[7] Dong K., Lu F., Huang W., Zhu L. (2020). Residual stress and fracture toughness of thick 8YSZ-Al_2_O_3_ composite coatings via a modified Vickers indentation method. Vacuum.

[8] Moulins A., Andrusyszyn F., Dugnani R., Zednik R.J. (2022). Indentation fracture toughness of semiconducting gallium arsenide at elevated temperatures. Eng. Fail. Anal..

[9] Li L., Wan L., Zhou Q. (2020). Crack propagation during Vickers indentation of zirconia ceramics. Ceram. Int..

[10] Rao X., Zhang F., Luo X., Ding F. (2019). Characterization of hardness, elastic modulus and fracture toughness of RB-SiC ceramics at elevated temperature by Vickers test. Mater. Sci. Eng..

[11] Begand S., Spintzyk S., Geis-Gerstorfer J., Bourauel C., Keilig L., Lohbauer U., Worpenberg C., Greuling A., Adjiski R., Jandt K.D., Lümkemann N. (2022). Fracture toughness of 3Y-TZP ceramic measured by the Chevron-Notch Beam method: a round-robin study. Dent. Mater..

[12] Lubauer J., Belli R., Lorey T., Max S., Lohbauer U., Zorzin J.I. (2022). A split-Chevron-Notched-Beam sandwich specimen for fracture toughness testing of bonded interfaces. J. Mech. Behav. Biomed. Mater..

[13] To T., Célarié F., Roux-Langlois C., Bazin A., Gueguen Y., Orain H., Le Fur M., Burgaud V., Rouxel T. (2018). Fracture toughness, fracture energy and slow crack growth of glass as investigated by the Single-Edge Precracked Beam (SEPB) and Chevron-Notched Beam (CNB) methods. Acta Mater..

[14] Gallo L.S.A., Célarié F., Bettini J., Rodrigues A.C.M., Rouxel T., Zanotto E.D. (2022). Fracture toughness and hardness of transparent MgO–Al_2_O_3_–SiO_2_ glass-ceramics. Ceram. Int..

[15] Qin X., Su H., Feng Y., Zhao H., Pham T.N. (2022). Fracture and deformation behaviors of saturated and dried single-edge notched beam sandstones under three-point bending based on DIC. Theor. Appl. Fract. Mech..

[16] Cui J., Gong Z., Lv M., Rao P. (2018). Effect of notch depth on fracture toughness of zirconia ceramics tested by SEVNB method. Ceram. Int..

[17] Zhou X.P., Yang H.Q., Zhang Y.X. (2009). Rate dependent critical strain energy density factor of Huanglong limestone. Theor. Appl. Fract. Mech..

[18] Fan K., Pastor J.Y., Ruiz-Hervias J., Gurauskis J., Baudin C. (2016). Determination of mechanical properties of Al_2_O_3_/Y-TZP ceramic composites: influence of testing method and residual stresses. Ceram. Int..

[19] Palacios T., Tarancón S., Abad C., Pastor Y.J. (2021). Saliva influence on the mechanical properties of advanced CAD/CAM composites for indirect dental restorations. Polymers.

[20] Ding J.C., Xu W. (2021). Determination of mode I interlaminar fracture toughness of composite by a wedge-insert double cantilever beam and the nonlinear J integral. Compos. Sci. Technol..

[21] Ponnusami S.A., Cui H., Erice B., Lißner M., Pathan M., Petrinic N. (2022). An integrated inverse numerical–experimental approach to determine the dynamic Mode-I interlaminar fracture toughness of fibre composites. Compos. Struct..

[22] Shi W., Zhang C., Wang B., Li M., Zhang C. (2023). Mode I interlaminar fracture toughness of two-dimensional continuous fiber reinforced ceramic matrix composites using wedge-loaded double cantilever beam method. Compos. Part A. Appl. Sci. Manuf..

[23] Lopes A.C., Silva E.C., Dourado N., Moura M.F.S.F., Sampaio A.M., Pontes A.J. (2022). The Double Cantilever Beam test applied to mode I fracture characterization of polyamide 12 processed by selective laser sintering technology. Eng. Fract. Mech..

[24] Mega M., Dolev O., Banks-Sills L. (2022). Fracture toughness resistance curves for a delamination in CFRP MD laminate composites, Part I: nearly mode II deformation. Theor. Appl. Fract. Mech..

[25] Pérez-Galmés M., Renart J., Sarrado C., Rodríguez-Bellido A., Costa J. (2016). A data reduction method based on the J-integral to obtain the interlaminar fracture toughness in a mode II end-loaded split (ELS) test. Compos. Part A. Appl. Sci. Manuf..

[26] Rickhey F., Marimuthu K.P., Lee J.H., Lee H., Hahn J.H. (2015). Evaluation of the fracture toughness of brittle hardening materials by Vickers indentation. Eng. Fract. Mech..

[27] Xu G.T., Luo J., Lu F.Q., Wang G., Liu H.T., Zhao M. (2022). Characterization of fracture toughness for surface-modified layer of 18CrNiMo7-6 alloy steel after carburizing heat treatment by indentation method. Eng. Fract. Mech..

[28] Gulivindala G., Karanam M.K., Ramadurai R., Chinthapenta V. (2022). Indentation based fracture toughness estimation of barium titanate thin film using experiments and simulations. Thin Solid Films.

[29] Mao W., Zhang H., Zhang Z., Xiong J., Huang H., Wang Y., Lv L., Feng B., Pan J., Dai C., Fang D. (2020). Evaluation of fracture toughness and residual stress for both single GYbZ and double layered GYbZ/8YSZ coatings by modified indentation tests. Surf. Coating. Technol..

[30] D'Silva G.J., Goanta V., Ciocanel C. (2021). Fracture toughness evaluation of Ni_2_MnGa magnetic shape memory alloys by Vickers micro indentation. Eng. Fract. Mech..

[31] Ghorbal G.B., Tricoteaux A., Thuault A., Louis G., Chicot D. (2017). Comparison of conventional Knoop and Vickers hardness of ceramic materials. J. Eur. Ceram. Soc..

[32] Ghorbal G.B., Tricoteaux A., Thuault A., Louis G., Chicot D. (2017). Mechanical characterization of brittle materials using instrumented indentation with Knoop indenter. Mech. Mater..

[33] Ghorbal G.B., Tricoteaux A., Thuault A., Ageorges H., Roudet F., Chicot D. (2020). Mechanical properties of thermally sprayed porous alumina coating by Vickers and Knoop indentation. Ceram. Int..

[34] Guo H., Jiang C.B., Yang B.J., Wang J.Q. (2018). On the fracture toughness of bulk metallic glasses under Berkovich nanoindentation. J. Non-Cryst..

[35] Yen C.Y., Jian S.R., Tseng Y.C., Juang J.Y. (2018). The deformation behavior and fracture toughness of single crystal YSZ (111) by indentation. J. Alloys Compd..

[36] Zhang D., Sun Y., Gao C., Liu M. (2020). Measurement of fracture toughness of copper via constant-load microscratch with a spherical indenter. Wear.

[37] Gogotsi G.A. (2003). Fracture toughness of ceramics and ceramic composites. Ceram. Int..

[38] Zawischa M., Makowski S., Kuczyk M., Weihnacht V. (2022). Comparison of fracture properties of different amorphous carbon coatings using the scratch test and indentation failure method. Surf. Coating. Technol..

[39] Samantaray B.K., Bakshi S.R., Rajulapati K.V., Gollapudi S. (2022). Hardness and indentation fracture toughness in a novel silicon composite synthesized by spark plasma sintering. Metal. Mater. Trans. A..

[40] Li C., Ding J., Zhang L., Wu C., Sun L., Lin Q., Liu Y., Jiang Z. (2022). Densification effects on the fracture in fused silica under Vickers indentation. Ceram. Int..

[41] Yaşar Z.A., Celik A.M., Haber R.A. (2022). Improving fracture toughness of B_4_C–SiC composites by TiB_2_ addition. Int. J. Refract. Met. Hard Mater..

[42] Abbas S.Z., Khalid F.A., Zaigham H. (2017). Indentation fracture toughness behavior of FeCo-based bulk metallic glass intrinsic composites. J. Non-Cryst..

[43] Fischer H., Marx R. (2002). Fracture toughness of dental ceramics: comparison of bending and indentation method. Dent. Mater..

[44] Mikowski A., Serbena F.C., Foerster C.E., Jurelo A.R., Maurício Lepienski C. (2011). A method to measure fracture toughness using indentation in REBa_2_Cu_3_O_7-δ_ superconductor single crystals. J. Appl. Phys..

[45] Amiri S., Lecis N., Manes A., Giglio M. (2014). A study of a micro-indentation technique for estimating the fracture toughness of Al6061-T6. Mech. Res. Comms..

[46] Liu H., Zhang J., Zhao M., Lu C. (2022). Determination of the fracture toughness of glasses via scratch tests with a Vickers indenter. Acta Mech. Solida Sin..

[47] Moradkhani A., Baharvandi H., Tajdari M., Latifi H., Martikainen J. (2013). Determination of fracture toughness using the area of micro-crack tracks left in brittle materials by Vickers indentation test. J. Adv. Ceram..

[48] Palmqvist S. (1957). Jernkontorets Ann.

[49] Evans A., Charles E. (1976). Fracture toughness determinations by indentation. J. Am. Ceram. Soc..

[50] Marshall D.B., Lawn B.R. (1986). Indentation of brittle materials. Microindentation Tech. Mater. Sci. Eng. ASTM STP..

[51] Armstrong R.W., Elban W.L. (1985). Dislocation aspects of plastic flow and cracking at indentations in magnesium oxide and cyclotrimethylnetrinitramine explosive crystals. Microindentation Tech. Mater. Sci. Eng. ASTM Int..

[52] Nastic A., Merati A., Bielawski M., Bolduc M., Fakolujo O., Nganbe M. (2015). Instrumented and Vickers indentation for the characterization of stiffness, hardness and toughness of zirconia toughened Al_2_O_3_ and SiC armor. J. Mater. Sci. Technol..

[53] Palmqvist S. (1962).

[54] Shetty D.K., Wright I.G., Mincer P.N., Clauer A.H. (1985). Indentation fracture of WC-Co cermets. J. Mater. Sci..

[55] Evans A.G. (1979).

[56] Jis R. (1990).

[57] Lankford J. (1982). Indentation microfracture in the Palmqvist crack regime: implications for fracture toughness evaluation by the indentation method. J. Mater. Sci. Lett..

[58] Cui C., Wang F., Hu Z., Li Y., Jiao B., Xue J., Wu M., Zhou L., Zhang W. (2021). Estimation of fracture toughness for ε zirconium hydride by Vickers micro-hardness indentation method. J. Met..

[59] Zacharie-Aubrun I., Henry R., Blay T., Brunaud L., Gatt J.M., Noirot J., Meille S. (2021). Effects of irradiation on mechanical properties of nuclear UO_2_ fuels evaluated by Vickers indentation at room temperature. J. Nucl. Mater..

[60] Fals H.C., Lourençato L.A., Orozco M.S., Belém M.J.X., Lima C.R.C. (2020). Slurry erosion resistance of thermally sprayed Nb_2_O_5_ and Nb_2_O_5_+WC12Co composite coatings deposited on AISI 1020 carbon steel. Ceram. Int..

[61] Ma D.J., Sun L., Wang L.Z., Wang J.L. (2018). A new formula for evaluating indentation toughness in ceramics. Exp. Mech..

[62] Guo Y., Li J., Zhang Y., Feng S., Sun H. (2021). High-entropy R_2_O_3_-Y_2_O_3_-TiO_2_-ZrO_2_-Al_2_O_3_ glasses with ultrahigh hardness, Young's modulus, and indentation fracture toughness. iscience.

[63] Gross T.M., Liu H., Zhai Y., Huang L., Wu J. (2020). The impact of densification on indentation fracture toughness measurements. J. Am. Ceram. Soc..

[64] ASTM C769-98 (2005). Standard test method for sonic velocity in manufactured carbon and graphite materials for use in obtaining an approximate Young's modulus, developed by subcommittee: D02.F0. Book of Standards.

[65] Toktaş G., Korkmaz S. (2023). Indentation fracture toughness of boronized unalloyed and alloyed ductile iron. Mater. Chem. Phys..

[66] Fan X., Luo Z., Mao W., Deng X., Ye W., Dai C. (2023). Effect of heat treatment on hardness and fracture toughness of Si/BSAS/Yb_2_SiO_5_ EBCs system under 1200° C. Ceram. Int..

[67] Lai S., Zang J., Shen W., Huang G., Fang C., Zhang Y., Chen L., Wang Q., Wan B., Jia X., Zhang Z. (2023). High hardness and high fracture toughness B_4_C-diamond ceramics obtained by high-pressure sintering. J. Eur. Ceram. Soc..

[68] Niihara K. (1983). A fracture mechanics analysis of indentation-induced Palmqvist crack in ceramics. J. Mater. Sci. Lett..

[69] Lawn B.R., Fuller E.R. (1975). Equilibrium penny-like cracks in indentation fracture. J. Mater. Sci..

[70] Evans A.G., Wilshaw T.R. (1976). Quasi-static solid particle damage in brittle solids—I. Observations analysis and implications. Acta Metall..

[71] Laugier M.T. (1987). New formula for indentation toughness in ceramics. J. Mater. Sci. Lett..

[72] Anstis G.R., Chantikul P., Lawn B.R., Marshall D.B. (1981). A critical evaluation of indentation techniques for measuring fracture toughness: I, direct crack measurements. J. Am. Ceram. Soc..

[73] Lawn B.R., Evans A.G., Marshall D.B. (1980). Elastic/plastic indentation damage in ceramics: the median/radial crack system. J. Am. Ceram. Soc..

[74] Feng Y., Zhang T., Yang R. (2011). A work approach to determine Vickers indentation fracture toughness. J. Am. Ceram. Soc..

[75] Chicot D., Duarte G., Tricoteaux A., Jorgowski B., Leriche A., Lesage J. (2009). Vickers indentation fracture (VIF) modeling to analyze multi-cracking toughness of titania, alumina and zirconia plasma sprayed coatings,”. Mater. Sci. Eng..

[76] Jamshidian M., Promoppatum P., Ramamurty U., Jhon M.H. (2022). Modulating fracture toughness through processing-mediated mesostructure in additively manufactured Al-12Si alloy. Mater. Des..

[77] Okuma G., Maeda K., Yoshida S., Takeuchi A., Wakai F. (2022). Morphology of subsurface cracks in glass-ceramics induced by Vickers indentation observed by synchrotron X-ray multiscale tomography. Sci. Rep..

[78] Gu Y.C. (2015).

[79] Latifi H., Moradkhani A., Baharvandi H., Martikainen J. (2014). Fracture toughness determination and microstructure investigation of a B_4_C–nanoTiB_2_ composite with various volume percent of Fe and Ni additives. Mater. Des..

[80] Moradkhani A., Baharvandi H. (2017). Determining the fracture resistance of B_4_C-nanoSiB_6_ nanocomposite by Vickers indentation method and exploring its mechanical properties. Int. J. Refract. Met. Hard Mater..

[81] Moradkhani A., Baharvandi H., Samani M. (2016). Mechanical properties and microstructure of B_4_C–nanoTiB_2_–Fe/Ni composites under different sintering temperatures. Mater. Sci. Eng..

[82] Moradkhani A., Baharvandi H., Naserifar A. (2019). Effect of sintering temperature on the grain size and mechanical properties of Al_2_O_3_-SiC nanocomposites. J. Korean Ceram. Soc..

[83] Moradkhani A., Baharvandi H. (2017). Microstructural analysis of fracture surfaces and determination of mechanical properties of Al_2_O_3_–SiC–MgO nanocomposites. Int. J. Refract. Met. Hard Mater..

[84] Moradkhani A., Baharvandi H. (2018). Mechanical properties and fracture behavior of B_4_C-nano/micro SiC composites produced by pressureless sintering. Int. J. Refract. Met. Hard Mater..

[85] Moradkhani A., Baharvandi H., Naserifar A. (2019). Fracture toughness of 3Y-TZP dental ceramics by using vickers indentation fracture and SELNB methods. J. Korean Ceram. Soc..

[86] Moradkhani A., Baharvandi H. (2018). Effects of additive amount, testing method, fabrication process and sintering temperature on the mechanical properties of Al_2_O_3_/3Y-TZP composites. Eng. Fract. Mech..

[87] Moradkhani A., Baharvandi H. (2018). Analyzing the microstructures of W-ZrC composites fabricated through reaction sintering and determining their fracture toughness values by using the SENB and VIF methods. Eng. Fract. Mech..

[88] Spangenberger A.G., Lados D. (2015). Integrated experimental, analytical, and computational design for fatigue crack growth resistance in cast aluminum alloys. Process Eng..

[89] Kozekanan B.S., Moradkhani A., Baharvandi H., Ehsani N. (2021). Mechanical properties of SiC-C-B_4_C composites with different carbon additives produced by pressureless sintering. Int. J. Appl. Ceram. Technol..

[90] Baharvandi H., Tajdari M., Moradkhani A. (2015). Study of fracture toughness in B_4_C-TiB_2_ nanocomposites with Vickers indentation test method at different loads. Amirkabir J. Sci. Res..

[91] Bobaru F., Foster J.T., Geubelle P.H., Silling S.A. (2016).

[92] Silling S.A. (2023). 2023 Joint Mathematics Meetings.

[93] Can U., Silling S.A., Guven I. (2023). A peridynamic investigation of ceramic material response under high-speed solid impact loadings. AIAA SCITECH 2023 Forum.

[94] Wu L., Huang D., Bobaru F. (2021). A reformulated rate-dependent visco-elastic model for dynamic deformation and fracture of PMMA with peridynamics. Int. J. Impact Eng..

[95] Mehrmashhadi J., Bahadori M., Bobaru F. (2020). On validating peridynamic models and a phase-field model for dynamic brittle fracture in glass. Eng. Fract. Mech..

[96] Bobaru F. (2007). Influence of van der Waals forces on increasing the strength and toughness in dynamic fracture of nanofibre networks: a peridynamic approach. Model. Simulat. Mater. Sci. Eng..

[97] Askari E., Bobaru F., Lehoucq R.B., Parks M.L., Silling S.A., Weckner O. (2008). Peridynamics for multiscale materials modeling. J. Phys. Conf..

[98] Silling S.A., Bobaru F. (2005). Peridynamic modeling of membranes and fibers. Int. J. Non Lin. Mech..

[99] Oterkus E., Diyaroglu C., Zhu N., Oterkus S., Madenci E. (2013). Nanopackaging: from Nanomaterials to the Atomic Scale: Proceedings of the 1^st^ International Workshop on Nanopackaging.

[100] Fan Y., You H., Tian X., Yang X., Li X., Prakash N., Yu Y. (2022). A meshfree peridynamic model for brittle fracture in randomly heterogeneous materials. Comput. Methods Appl. Mech. Eng..

[101] Ignatiev M.O., Petrov Y.V., Kazarinov N.A., Oterkus E. (2022). Peridynamic formulation of the mean stress and incubation time fracture criteria and its correspondence to the classical Griffith's approach. Continuum Mech. Therm..

